# Digitized Acoustic Analysis for Monitoring Hemodialysis Access Dysfunction: Insights from Vascular Imaging and Post-Angioplasty Data

**DOI:** 10.3390/jcm15020662

**Published:** 2026-01-14

**Authors:** Hsien-Yuan Chang, Yi-Ling Kuo, Christian Deantana, Chih-Chang Ko, Po-Wei Chen, Tsai-Chieh Ling, Che-Wei Lin, Kun-Chan Lan

**Affiliations:** 1 Division of Cardiology, Department of Internal Medicine, National Cheng Kung University Hospital, College of Medicine, National Cheng Kung University, Tainan 704, Taiwan; doyeric0926@yahoo.com (H.-Y.C.);; 2 Institute of Clinical Medicine, College of Medicine, National Cheng Kung University, Tainan 704, Taiwan; 3 Department of Computer Science and Information Engineering, National Cheng Kung University, Tainan 704, Taiwan; 4 Division of Nephrology, Department of Internal Medicine, National Cheng Kung University Hospital, College of Medicine, National Cheng Kung University, Tainan 704, Taiwan; 5 Department of Biomedical Engineering, College of Engineering, National Cheng Kung University, Tainan 704, Taiwan

**Keywords:** hemodialysis vascular access dysfunction, angioplasty, acoustic analysis, phono-angiography, sound analysis

## Abstract

**Background:** Hemodialysis access dysfunction can lead to missed treatments and increased mortality. Traditional monitoring methods, such as physical examination and ultrasound, have limitations, emphasizing the need for a more efficient approach. This study investigates the use of digitized acoustic data to identify and monitor vascular access dysfunction. **Methods:** This prospective study involved patients undergoing hemodialysis with either arteriovenous fistulas (AVF) or arteriovenous grafts (AVG) between June 2023 and February 2025. All patients underwent vascular imaging (either angiography or ultrasound) to confirm the degree of stenosis. Acoustic data were recorded using a standardized procedure at various puncture sites. Pre- and post-angioplasty data were also collected to assess the effects of vascular intervention. The digitized acoustic data were analyzed for changes in relative loudness, peak-to-valley ratios, and frequency distribution. **Results:** A total of 157 patients with 236 audio recordings (mean age: 67 ± 11 years; 58% male) were included. Significant acoustic differences were found at the arterial puncture and anastomosis sites in AVF patients with dysfunction, particularly in venous site dysfunction, which exhibited a more pronounced reduction in sound volume and an increased peak-to-valley ratio. After angioplasty, acoustic changes were observed in both arterial and venous sites, with values moving toward normal levels. However, no significant acoustic changes were observed in AVG patients. Additionally, frequency distribution ratios showed minimal clinical relevance. **Conclusions:** Digitized acoustic data, particularly from the arterial puncture and anastomosis sites, can be an effective tool for detecting and monitoring hemodialysis access dysfunction. These findings suggest potential for acoustic analysis in clinical practice, especially when integrated with AI models for better diagnostics.

## 1. Introduction

Hemodialysis vascular access dysfunction is a worrisome complication that can result in missed dialysis sessions, inpatient admission, the need for temporary catheter placement, and even increased mortality [1]. Early detection of hemodialysis access dysfunction remains a critical clinical challenge. In addition to reduced intra-access flow or elevated venous outflow pressure during hemodialysis, current methods available for monitoring and surveillance of hemodialysis access include physical examination, duplex ultrasound imaging, angiography, and other indirect techniques [2]. Although physical examination is the most convenient method, it requires a trained professional. Duplex ultrasound, while widely used, is operator-dependent and subject to variability based on the chosen insonation angle. Other methods either require specialized equipment or are time-consuming. Thus, there is a clear need for a quick and efficient assessment method that can be performed before dialysis therapy or even in home care settings.

Vascular sound analysis represents a convenient, non-invasive, and potentially low-cost method for monitoring hemodialysis access function, which could be applied at the bedside or even in home care settings. Historically, its development has been limited by the challenges of systematically analyzing, quantifying, and interpreting acoustic signals. Recent advances in deep learning and signal processing have made automated and more accurate analyses feasible, offering the potential for earlier, more convenient, and objective detection of vascular access dysfunction compared with traditional methods. For example, phonocardiography, the analysis of heart sounds, has been successfully employed in detecting valvular diseases [3]. Similarly, the application of sound analysis to detect vascular stenosis, termed phono-angiography, is an emerging area with the potential to develop into a convenient, accessible, fast, non-invasive, and low-cost method for detecting hemodialysis access dysfunction [4,5,6,7,8,9]. Various devices, including prototype models [10] and microphone arrays [11], are currently under development.

Unlike valvular diseases, which have standardized diagnostic criteria and sound recording positions, there is no standardized protocol for capturing sounds related to vascular access dysfunction. The heterogeneity of arteriovenous fistula types, vessel size, orientations, locations, causes of dysfunction, and the presence of collateral flow may all influence the results. Current sound models for detecting vascular access dysfunction often rely on a stenosis threshold greater than 50% to classify lesions, but they do not always account for the clinical functional status of the fistula. Incorporating clinical dysfunction criteria, such as suction alarms or elevated venous pressure, in addition to anatomical stenosis, could potentially improve clinical relevance. A physiological understanding of how different types of dysfunctions affect acoustic parameters is also crucial for guiding future artificial intelligence-based predictive models.

To bridge the gap between deep learning models and clinical practice, pioneering research that explores sound information from a physiological perspective in hemodialysis access dysfunction patients is essential. In this study, we aim to standardize the process of sound recording in patients with vascular access dysfunction and investigate the significance of various sound parameters in identifying the types of dysfunctions. Additionally, we will examine the differences before and after vascular interventions from a clinical perspective.

## 2. Methods

### 2.1. Study Design

This was a prospective study that collected acoustic data of hemodialysis vascular access from patients between 20 June 2023 and 20 February 2025. The study adhered to the principles outlined in the Declaration of Helsinki and received approval from the Human Research and Ethics Committee of National Cheng Kung University Hospital (IRB number: B-ER-112-164). Written informed consent was obtained from all enrolled patients.

### 2.2. Study Population

To investigate the relationship between acoustic data and the degree of stenosis, we enrolled patients using arteriovenous fistulas (AVF) or arteriovenous grafts (AVG) as vascular access for hemodialysis who had also undergone either angiography or ultrasound. Patients under 20 years of age and those who did not provide written informed consent were excluded from the study. Patients who underwent only ultrasound examinations were classified as the normal group based on the judgment of the attending physician. For patients who underwent angiography, those with a stenosis degree of less than 50% who were clinically non-dysfunctional and did not require percutaneous transluminal angioplasty (PTA) were classified as the normal group. This classification aligns with standard clinical practice. Dysfunctional fistulas were defined by attending physicians in the dialysis clinic based on clinical criteria. Most cases were identified due to suction alarms or persistently elevated venous pressure during dialysis; additional indicators included abnormal findings on palpation or auscultation and occasionally decreased dialysis adequacy. Patients were subsequently categorized as having arterial- or venous site dysfunction according to their presenting symptoms and angiographic findings. In general, dysfunction associated primarily with suction was considered arterial site, and dysfunction associated with elevated venous pressure was considered venous site, while cases with atypical or overlapping features were classified based on the attending physician’s overall clinical judgment. Patients were first grouped by access type (AVF or AVG). For AVF cases, dysfunction was further classified as arterial site or venous site based on clinical presentation and angiographic findings. Clinical information, including age, sex, and type of vascular access (AVF or AVG), was recorded.

### 2.3. Standardized Acoustic Information Collection

Acoustic information will be obtained by using Stemoscope—Smart Listening Device (Shanghai Hulu, Shanghai, China), a non-medical device designed for general acoustic analysis. Prior to data collection, we evaluated several factors that might affect the acoustic signals. A 25 g calibration weight did not significantly impact the acoustic data compared to using paper glue or straps, whereas hand pressure was found to influence the recordings. The effect of surrounding noise was also assessed, but no significant changes to the waveform quality were observed. Based on these findings, the data collection procedure was standardized: the patient’s hand was placed flat on a table, and the device was secured with straps, ensuring a two-finger-width gap between the strap and the arm to avoid compression.

Due to the heterogeneity of fistulas and the need for reproducible and standardized acoustic measurements, we selected puncture sites as reference points for data collection. For AVF patients, acoustic data were collected at the arterial anastomosis and at arterial and venous puncture sites, which correspond to the usual cannulation points. For AVG patients, data were collected at arterial and venous puncture sites. Using puncture sites as consistent landmarks ensures comparability of acoustic signals across patients despite anatomical variability. Each location was recorded for 10–20 s. The frequency range of the collected audio was selected from 20 to 1000 Hz. The recorded sound was in digital waveform format, displayed as relative loudness with values ranging from −32,768 to 32,767. In patients who underwent angioplasty, acoustic data were collected both pre- and post-PTA on the same day.

### 2.4. Angiography

The location and diameter of stenosis were reviewed by two specialists based on angiography images. Arterial site dysfunction was defined as PTA performed on the distal vessel of the arterial puncture site, while venous site dysfunction was defined as PTA performed on the proximal vessel of the venous puncture site. The diameter of stenosis was measured as the inner lumen diameter, and the percentage of stenosis was calculated as the ratio of the stenotic diameter to the diameter of the adjacent normal vessel.

### 2.5. Digitized Acoustic Data

**Figure 1:**
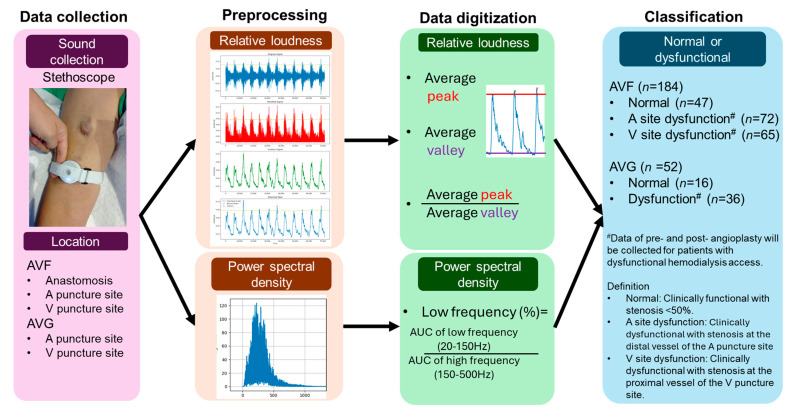
Flowchart of the study cohort. (Abbreviations: A: Arterial; AUC: Area Under the Curve; AVF: Arteriovenous Fistula; AVG: Arteriovenous Graft; V: Venous.).

Since the pulsation and thrill of fistulas differ between the systolic and diastolic phases, these variations are useful in the physical examination of hemodialysis access to identify the location of dysfunctional access. Therefore, we separately digitized the relative loudness during the systolic and diastolic phases, as well as the frequency distribution ratios (Figure 1).

### 2.6. Preprocessing

#### 2.6.1. Bandpass Filtering

To isolate the relevant frequency components of the signal, a bandpass filter is applied to each audio signal. We selected a 20–500 Hz bandpass because vascular access bruits predominantly contain energy at lower frequencies related to turbulent blood flow, with studies showing peak spectral power around ~200 Hz and useful components concentrated below ~500 Hz, while very low frequencies (<20 Hz) are dominated by motion or baseline artifacts [12]. The filter preserves frequencies between 20 Hz and 500 Hz while attenuating the remaining frequencies.

#### 2.6.2. Rectification

After bandpass filtering, the audio signal is rectified to eliminate negative values, ensuring that all signal amplitudes are positive. This is achieved by taking the absolute value of the filtered signal, which enhances relevant components, such as peak detection.

#### 2.6.3. Generalized Contour Extraction

To extract a smooth contour, a rolling mean window of 500 samples is applied to the rectified signal, reducing high-frequency fluctuations and providing a clearer trend representation. This smoothing process reduces high-frequency fluctuations, providing a clearer representation of the underlying trend in the signal.

#### 2.6.4. Normalization

Normalization is performed to standardize the amplitude of the generalized contour signal. The 99th percentile value of the original audio signal and the 90th percentile of the generalized contour signal are used for normalization.

#### 2.6.5. Peak/Valley Detection

To identify features of peaks and valleys in the generalized contour signal, a peak/valley detection algorithm is applied. The signal.find_peaks function in SciPy library is used [13], with a minimum distance of 1000 samples between detected peaks/valleys to prevent the identification of closely spaced peaks as separate events.

#### 2.6.6. Outlier Detection and Statistical Analysis

Detected peaks and valleys are further analyzed to remove outliers based on the interquartile range (IQR) of the peak/valley amplitudes. The conventional 1.5 × IQR rule is a heuristic and can be adjusted to suit specific noise characteristics of the data [14]. In our audio signal application, minor amplitude fluctuations from noise or measurement variability can lie well within the 1.5 × IQR bounds yet still distort feature estimates. By using a stricter 0.1 × IQR threshold, we more aggressively suppress these small, non-physiological variations while retaining genuine peak/valley structure. Peaks and valleys falling outside this range are considered outliers, and only the valid peaks and valleys are used to calculate the average amplitude.

#### 2.6.7. Frequency Band Calculation

The raw audio signals undergo processing similar to that used in the peak and valley detection method. Starting from the first detected valley, a 5 s segment of the signal is selected to calculate the area under the frequency spectrum. Two frequency bands are analyzed: the low band (20–150 Hz), which corresponds to the fundamental rhythm of blood flow, primarily reflecting the heartbeat [13], and the mid-to-high band (150–500 Hz), which represents turbulence caused by irregular or disrupted blood flow. The area under the curve (AUC) for each frequency band is calculated, and the low-frequency component (%) is then calculated as the ratio of the AUC of the low band to the total AUC. This approach helps characterize the acoustic features of both normal and turbulent blood flow.

### 2.7. Statistical Analysis

Statistical analysis was performed using SPSS version 21.0 (IBM, Armonk, NY, USA). Continuous data are presented as the mean ± standard deviation when normally distributed and as median with interquartile range when non-normally distributed. Dichotomous data are presented as numbers and percentages. Comparisons were conducted using Student’s *t*-test for normally distributed continuous variables, the Mann–Whitney U test for non-normally distributed continuous variables, and the chi-square test for categorical variables. For repeated measurements before and after PTA at each recording site, the paired *t*-test was used for normally distributed data, whereas the Wilcoxon signed-rank test was used for non-normally distributed data. Multiple sites per patient were analyzed independently. A *p*-value < 0.05 was considered to indicate statistical significance.

## 3. Results

### 3.1. Study Enrollment and Grouping

A total of 157 patients with 236 audio recordings (mean age: 67 ± 11 years; 58% male) were included in the study. Among these, 184 recordings were from AVF patients and 52 from AVG patients. For AVF patients, 47 were classified as normal, 72 as arterial site dysfunction, and 65 as venous site dysfunction, with stenosis degrees of 61 ± 12% and 68 ± 12%, respectively. For AVG patients, 36 were classified as dysfunctional and 16 as normal, with a stenosis degree of 65 ± 11%. There were no significant differences in age or sex between the groups (Figure 1) (Table 1).

### 3.2. Acoustic Data of Dysfunctional Vascular Access

The digitized acoustic data revealed significant differences in the anastomosis and arterial puncture sites among patients with AVF, showing a similar trend across groups. In contrast, the venous puncture site displayed less significant differences between the groups. This suggests that there are notable variations in acoustic data from different locations. Therefore, detection protocols for dysfunctional fistulas should ideally focus on the anastomosis or arterial puncture sites. Unfortunately, in patients with AVG, there was no significant difference in acoustic data between functional and dysfunctional fistulas.

**Figure 2:**
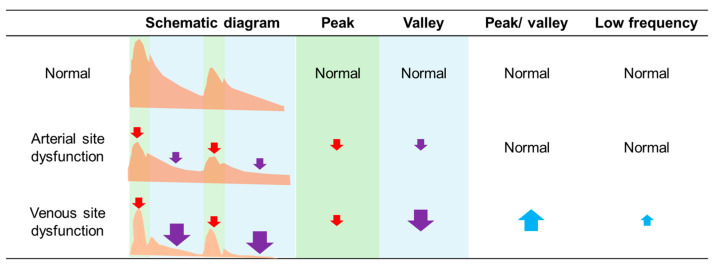
Summary of the main findings and changes in digitized sound data resulting from stenosis at different sites of the hemodialysis access. (Note: The green area represents the systolic phase, and the blue area represents the diastolic phase. Red arrows denote changes in peak values, purple arrows denote changes in valley values, and blue arrows denote changes in peak-to-valley differences. Upward arrows indicate an increase, downward arrows indicate a decrease. Arrow size represents the magnitude of change relative to the normal group, with larger arrows indicating greater changes.).

Taking the arterial puncture site as an example, the relative loudness of the peak and valley in normal AVF was 0.46 (0.21–0.67) and 0.09 (0.03–0.14), respectively. In patients with arterial site dysfunction, these values were significantly reduced (0.18 (0.09–0.48), *p* < 0.01; 0.03 (0.01–0.09), *p* < 0.01), and similarly, in patients with venous site dysfunction, the relative loudness also decreased significantly (0.17 (0.07–0.30), *p* < 0.01; 0.01 (0.01–0.04), *p* < 0.01, Table 1). Drawing parallels with changes in pulsation and thrill during AVF physical examinations, we also explored the ratio of peak to valley changes. We found that compared to patients with arterial site dysfunction, those with venous site dysfunction exhibited a significant increase in the peak/valley ratio (8.5 (4.8–12.7) vs. 4.8 (4.0–7.0), *p* < 0.01). Overall, while the loudness of the dysfunctional fistula’s sound decreased, the reduction in the diastolic phase was more pronounced in venous site dysfunction, leading to a significant increase in the peak/valley ratio (Figure 2). Our data suggests that the peak/valley ratio can be used to differentiate between arterial and venous site dysfunction.

As for frequency distribution ratios, although the venous site dysfunction group had significantly higher low frequencies, the difference at the anastomosis site was inconsistent and became non-significant. While frequency distribution ratios may enhance predictive accuracy, considering the difficulty of data acquisition and ease of interpretation, using relative loudness may be a simpler approach.

### 3.3. Changes in Acoustic Data After Angioplasty

After PTA, the vessel diameter significantly increased from an average of 2.7–2.9 mm to 5.0–5.6 mm, while the percentage of stenosis significantly decreased from 60–68% to 27–37%. Similarly, the changes in acoustic data were primarily observed at the arterial puncture site and anastomosis. In patients with venous site dysfunction, both the peak and valley values significantly increased after PTA, while the peak/valley ratio significantly decreased (Table 2). These changes moved toward the values observed in the normal group, further validating our earlier findings. On the other hand, in patients with arterial site dysfunction, the peak and valley values at the anastomosis site also significantly increased following PTA, with values approaching those of the normal group, supporting our previous observations. However, a notable exception was the arterial puncture site, as its peak value did not increase and its valley value even significantly decreased (0.03 (0.01–0.09) vs. 0.02 (0.02–0.06), *p* = 0.03). Additionally, for AVG patients, no significant changes were observed in the acoustic data. Furthermore, frequency distribution ratios showed no significant changes in any of the patients.

## 4. Discussion

The objective of this study was to standardize the process of auscultating hemodialysis access sounds and to digitize these acoustic data in order to compare the acoustic characteristics of dysfunctional vascular access and the changes observed before and after angioplasty. Our findings revealed that the acoustic differences associated with dysfunctional AVF were more pronounced near the arterial site. Overall, the loudness of the sound decreased in dysfunctional AVFs. Notably, when dysfunction occurred at the venous site, the sound volume during the vascular diastolic phase decreased to a greater extent compared to dysfunction at the arterial site. This led to a significant increase in the peak-to-valley ratio. Additionally, these differences largely diminished following PTA. Our results suggest that digitizing acoustic data from hemodialysis access can be effectively applied to clinical assessments for identifying vascular access dysfunction.

To facilitate the subsequent comparison of digitized acoustic data, we first standardized the process for recording sounds. Previous studies have used various reference points for sound measurement, such as 1–2 cm distal to the anastomosis site [8], five fixed intervals [7], wearable devices [15], thermal sensors [16] and puncture sites [9]. For practical reasons related to both patients and clinical staff, and to ensure standardized and reproducible measurements, we focused on the puncture sites and anastomosis. Recent pilot studies employing digital auscultation have similarly highlighted that selecting easily identifiable anatomical landmarks improves consistency across subjects [17]. These locations serve as reliable landmarks that facilitate consistent acoustic data collection across different individuals. On the other hand, we initially compared the use of a 25 g calibration weight, 3M paper glue, and light manual fixation and found that light manual pressure influenced the acoustic signals. To minimize the interference caused by pressure, we decided to secure the device using straps, ensuring a two-finger-width gap between the strap and the arm to prevent compression. Standardized acquisition facilitates downstream quantitative and machine learning analyses, as demonstrated in recent deep learning studies on vascular access sounds [18]. Additionally, to ensure accurate diagnosis of access dysfunction and stenosis, all participants underwent either angiography or vascular ultrasound. To further reduce selection bias, we also included both pre- and post-PTA acoustic data from patients undergoing vascular interventions, allowing us to better validate the differences in acoustic data related to dysfunctional vascular access. Our data suggest that, among the easier-to-locate sites, the arterial puncture site provides the most significant acoustic data, which could offer meaningful insights for the development of future protocols.

**Figure 3:**
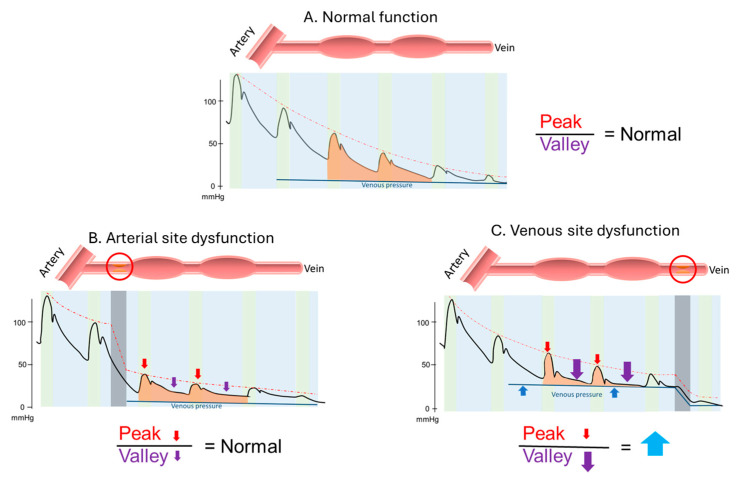
Schematic diagram of the theoretical changes in peak, valley, and peak/valley ratio due to pressure gradients at different stenotic sites. (**A**) Normal function. (**B**) Dysfunction at the arterial site. (**C**) Dysfunction at the venous site. (Note: The upper diagram illustrates stenosis at the arteriovenous fistula, with the red circle indicating the stenotic site. The lower diagram schematically depicts pressure changes at the corresponding locations. The green area represents the systolic phase, and the blue area represents the diastolic phase. The colored areas represent changes during systole and diastole, not locations, and are shown together for illustrative purposes. Red arrows denote changes in peak values, purple arrows denote changes in valley values, and blue arrows denote changes in peak-to-valley differences. Upward arrows indicate an increase, downward arrows indicate a decrease. Arrow size represents the magnitude of change relative to the normal group, with larger arrows indicating greater changes.).

**Figure 4:**
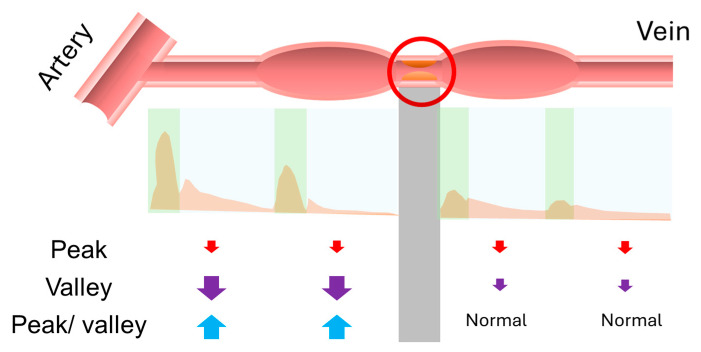
Schematic diagram showing the application of peak/valley changes to detect the location of stenosis. (Note: The upper diagram illustrates stenosis at the arteriovenous fistula, with the red circle indicating the stenotic site. The middle diagram shows the corresponding waveform inferred from this location. The green area represents the systolic phase, and the blue area represents the diastolic phase. The gray shaded area indicates the region corresponding to the stenotic site. The lower diagram schematically depicts changes in values at the corresponding locations. Red arrows denote changes in peak values, purple arrows denote changes in valley values, and blue arrows denote changes in peak-to-valley differences. Upward arrows indicate an increase, whereas downward arrows indicate a decrease. Arrow size represents the magnitude of change relative to the normal group, with larger arrows indicating greater changes.).

After undergoing a series of preprocessing steps, the acoustic data, similar to the pulsation and thrill in physical examination, were digitized by defining the peak and valley to represent the systolic and diastolic phases of the sound, respectively. We then calculated the ratio between the peak and valley to correspond to the ratio of pulsation and thrill. Our findings revealed that in dysfunctional AVF, the overall sound volume decreased, and when there was venous stenosis, the decrease in diastolic phase became more pronounced. This resulted in a significant increase in the peak-to-valley ratio, a finding that was also observed in the changes before and after vascular interventions. This change was partially validated by the numerical differences observed in the pre- and post-PTA data. Next, we explored the potential physiological significance of these findings. A stenosed vessel may generate a pressure gradient, with arterial stenosis potentially leading to an overall decrease in pressure, which could cause the sound to become quieter (Figure 3). Changes in frequency and amplitude patterns have been shown to correlate with hemodynamic conditions in AVFs, supporting the concept that sound features can reflect complex flow dynamics and pressure gradients [19]. On the other hand, the pressure difference at the venous site may increase the outflow resistance of the vessel, particularly reducing the diastolic pressure difference. The location of the pressure gradient could therefore influence the ratio of systolic to diastolic pressure differences. From this physiological perspective, the change in the peak-to-valley ratio is therefore plausible. Moreover, this observation may theoretically aid in localizing stenotic regions, similar to physical examination, where pulsation appears strong and thrill weak before a stenosis, and thrill increases after the stenosis. In the digitized acoustic data, the peak-to-valley ratio may be higher before the stenosis and lower after it. Therefore, theoretically, the position where the peak-to-valley ratio appears to change could indicate the location of the vascular stenosis (Figure 4). Our findings provide insights that could support clinical application of visualized [20] or digitized acoustic data in the detection of dysfunctional hemodialysis access, but further validation is needed.

Although most acoustic changes post-PTA aligned with our expectations, one notable exception was observed specifically in patients with arterial site dysfunction, where peak and valley values at the arterial puncture site decreased rather than increased. This may be explained by a reduction in turbulent flow following angioplasty—likely due to restored luminal patency and smoother flow dynamics in the previously stenotic segment. Since turbulent flow generates greater acoustic energy than laminar flow, its reduction could result in lower sound intensity despite structural improvement. Notably, these same patients exhibited increased acoustic values at the anastomosis site, reinforcing the concept of site-specific hemodynamic responses and the importance of interpreting acoustic signals in their local vascular context. Future studies are warranted to explore whether continued vascular adaptation, such as through grip-strength rehabilitation, may lead to progressive normalization of acoustic profiles beyond the immediate post-intervention period. On the other hand, it is unfortunate that changes in acoustic data were not significant in the AVG group. This may be attributed to the different resistance characteristics of the foreign materials used in AVG, small differences in vessel diameters, or variations in the fluid dynamics. Furthermore, although frequency distribution ratios showed significant differences in certain areas, the clinical application of these differences remains challenging. These ratios may serve as an adjunctive tool in clinical practice, but their direct application would be difficult without further support. However, they could potentially be integrated into artificial intelligence models for analysis in future studies. Lastly, we attempted to apply our findings clinically and identified several potential confounding factors. These included the presence of large collateral flow, which appeared to normalize the acoustic profile, and the presence of large aneurysms, which seemed to reduce sound volume. Further research is needed to explore these factors in detail for future clinical applications.

Our study has several limitations. First, the sample size was relatively small. Second, due to the high heterogeneity of hemodialysis access, numerous factors, such as collateral flow and aneurysms, could not be fully enumerated or compared in detail. Third, we used a non-medical acoustic device with default acquisition settings, which may limit the generalizability of our results. Four, subgroup analyses were based on commonly used clinical distinctions (AVF vs. AVG, arterial vs. venous), which are well established in the literature. While other classification schemes exist, they were not used due to limited sample size and lack of clear additional differentiation in our dataset. Future research should validate these findings using different devices and settings to enhance clinical applicability.

In conclusion, this study highlights the potential of digitized acoustic data in assessing dysfunctional hemodialysis access, particularly in AVF. Our findings show that acoustic changes, such as decreased sound volume and increased peak-to-valley ratios, are significant indicators of dysfunction, especially near the arterial puncture site. These changes were largely reversed after PTA. Despite the promising results, limitations such as small sample size, heterogeneity of hemodialysis access, and confounding factors like collateral flow and aneurysms require further investigation. Overall, this approach could be valuable in clinical practice, particularly when combined with artificial intelligence models for enhanced analysis.

## Figures and Tables

**Table 1:** Acoustic data of hemodialysis vascular access with different types and locations of dysfunction.

	Arteriovenous Fistula					Arteriovenous Graft		
	Normal	Arterial Site Dysfunction		Venous Site Dysfunction		Normal	Dysfunction	
	(*n* = 47)	(*n* = 72)	* *p*-Value	(*n* = 65)	* *p*-Value	(*n* =16)	(*n* = 36)	* *p*-Value
Age (years old)	66 ± 11	68 ± 12	0.34	66 ± 11	0.93	69 ± 8	71 ± 12	0.63
Sex (Male)	25 (53%)	46 (64%)	0.25	40 (62%)	0.37	9 (56%)	17 (47%)	0.55
Stenosis (%)		61 ± 12		68 ± 12			65 ± 11	
Arterial puncture site								
Peak	0.46 (0.21–0.67)	0.18 (0.09–0.48)	* <0.01	0.17 (0.07–0.30)	* <0.01	0.27 (0.05–0.43)	0.15 (0.05–0.31)	0.52
Valley	0.09 (0.03–0.14)	0.03 (0.01–0.09)	* <0.01	0.01 (0.01–0.04)	* <0.01	0.04 (0.01–0.08)	0.02 (0.01–0.05)	0.47
Peak/valley	5.0 (4.3–6.4)	4.8 (4.0–7.0)	0.74	8.5 (4.8–12.7)	* <0.01	5.5 (4.3–6.0)	6.3 (4.8–7.9)	0.20
Low Frequency (%)	39 (31–55)	38 (29–49)	0.25	53 (40–62)	* 0.01	36 (23–44)	30 (24–39)	0.61
Venous puncture site								
Peak	0.11 (0.05–0.30)	0.05 (0.02–0.12)	* <0.01	0.10 (0.05–0.24)	0.59	0.21 (0.03–0.30)	0.08 (0.04–0.16)	0.16
Valley	0.02 (0.01–0.06)	0.01 (0.00–0.02)	* <0.01	0.01 (0.01–0.04)	0.16	0.04 (0.12–0.07)	0.01 (0.01–0.03)	0.07
Peak/valley	4.9 (4.3–6.0)	4.9 (3.7–6.8)	0.79	5.9 (4.4–8.9)	* 0.03	4.4 (3.4–5.2)	5.3 (3.8–6.3)	0.10
Low Frequency (%)	53 (40–59)	52 (36–61)	0.99	47 (38–59)	0.38	29 (21–33)	28 (23–35)	0.81
Anastomosis								
Peak	0.27 (0.14–1.01)	0.09 (0.05–0.26)	* <0.01	0.18 (0.08–0.25)	* <0.01	N/A		
Valley	0.05 (0.02–0.27)	0.02 (0.01–0.04)	* <0.01	0.01 (0.00–0.03)	* <0.01			
Peak/valley	4.6 (3.6–5.7)	5.0 (3.9–7.1)	0.07	10.6 (6.3–18.8)	* <0.01			
Low Frequency (%)	33 (29–42)	29 (23–36)	* 0.02	35 (25–49)	0.42			

Data are presented as the mean ± SD, median (IQR), or *n* (%), as appropriate. * Compared with the normal function group.

**Table 2:** Changes in acoustic data of hemodialysis vascular access before and after vascular angioplasty.

	Arteriovenous Fistula						Graft
	Arterial Site Dysfunction (*n* = 72)			Venous Site Dysfunction (*n* = 65)			Dysfunction (*n* = 36)
	Pre-PTA	Post-PTA	*p*-Value	Pre-PTA	Post-PTA	*p*-Value	Post-PTA
Stenosis size (mm)	2.7 ± 0.8	5.0 ± 1.0	<0.01	2.7 ± 1.4	5.2 ± 1.8	<0.01	5.6 ± 2.7
Stenosis (%)	60 ± 13	27 ± 13	<0.01	68 ± 12	37 ± 13	<0.01	33 ± 16
Arterial puncture site							
Peak	0.18 (0.09–0.48)	0.18 (0.11–0.30)	0.27	0.17 (0.07–0.30)	0.18 (0.10–0.38)	* <0.01	0.15 (0.05–0.31)
Valley	0.03 (0.01–0.09)	0.02 (0.02–0.06)	* 0.03	0.01 (0.01–0.04)	0.02 (0.01–0.06)	* <0.01	0.02 (0.01–0.05)
Peak/valley	4.8 (4.0–7.0)	6.0 (4.2–7.7)	* 0.01	8.5 (4.8–12.7)	6.2 (4.9–11.8)	* 0.02	6.3 (4.8–7.9)
Low Frequency (%)	38 (29–49)	46 (37–58)	* <0.01	53 (40–62)	51 (40–63)	0.4	30 (24–39)
Venous puncture site							
Peak	0.05 (0.02–0.12)	0.07 (0.03–0.18)	0.21	0.10 (0.05–0.24)	0.11 (0.05–0.22)	0.87	0.08 (0.04–0.16)
Valley	0.01 (0.00–0.02)	0.01 (0.01–0.02)	0.21	0.01 (0.01–0.04)	0.01 (0.01–0.05)	0.43	0.01 (0.01–0.03)
Peak/valley	4.9 (3.7–6.8)	5.4 (4.1–8.2)	0.09	5.9 (4.4–8.9)	5.5 (4.4–8.6)	0.32	5.3 (3.8–6.3)
Low Frequency (%)	52 (36–61)	52 (40–63)	0.8	47 (38–59)	49 (38–65)	0.33	28 (23–35)
Anastomosis							
Peak	0.09 (0.05–0.26)	0.23 (0.06–0.50)	* <0.01	0.18 (0.08–0.25)	0.12 (0.05–0.28)	0.56	N/A
Valley	0.02 (0.01–0.04)	0.04 (0.01–0.09)	* <0.01	0.01 (0.00–0.03)	0.01 (0.01–0.03)	0.32	
Peak/valley	5.0 (3.9–7.1)	4.9 (4.2–7.2)	0.62	10.6 (6.3–18.8)	7.4 (4.4–13.9)	* <0.01	
Low Frequency (%)	29 (23–36)	28 (24–36)	0.35	35 (25–49)	39 (29–53)	0.45	

Data are presented as the mean ± SD or median (IQR) as appropriate. Abbreviation: PTA: percutaneous transluminal angioplasty. * *p* < 0.05 compared with Pre-PTA values.

## Data Availability

The authors declare that they had full access to all the study data. The authors assume complete responsibility for the integrity of the data and the accuracy of the data analysis.
